# Supplementary material for: Nitrogen isotopes reveal high NOx emissions from arid agricultural soils in the Salton Sea Air Basin
Source: Sci Rep. 2024 Nov 20;14:28725. doi: 10.1038/s41598-024-78361-y (PMC11579327; doi:10.1038/s41598-024-78361-y)
Supplement: Supplementary file 1 — Supplementary Material 1 [file 41598_2024_78361_MOESM1_ESM.docx]

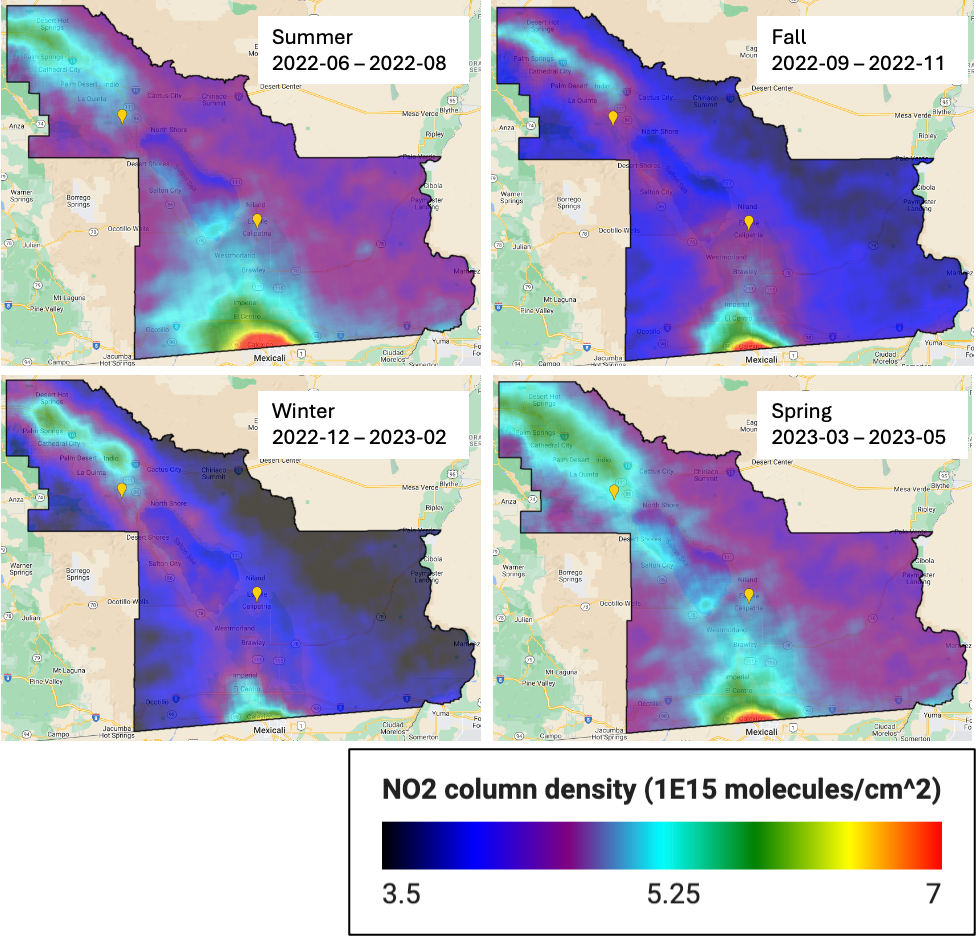


**Figure S1**. Seasonal average NO_2_ columns for the Salton Sea Air Basin during our sampling campaign (June 2022 – May 2023). Yellow points indicate the two sampling sites Calipatria (SE) and Thermal (NW). The map was created in Google Earth Engine using the Sentinel-5P NRTI NO_2_: Near Real-Time Nitrogen Dioxide dataset; the script to access this data was modified but the original script can be found at <https://developers.google.com/earth-engine/datasets/catalog/COPERNICUS_S5P_NRTI_L3_NO2>.


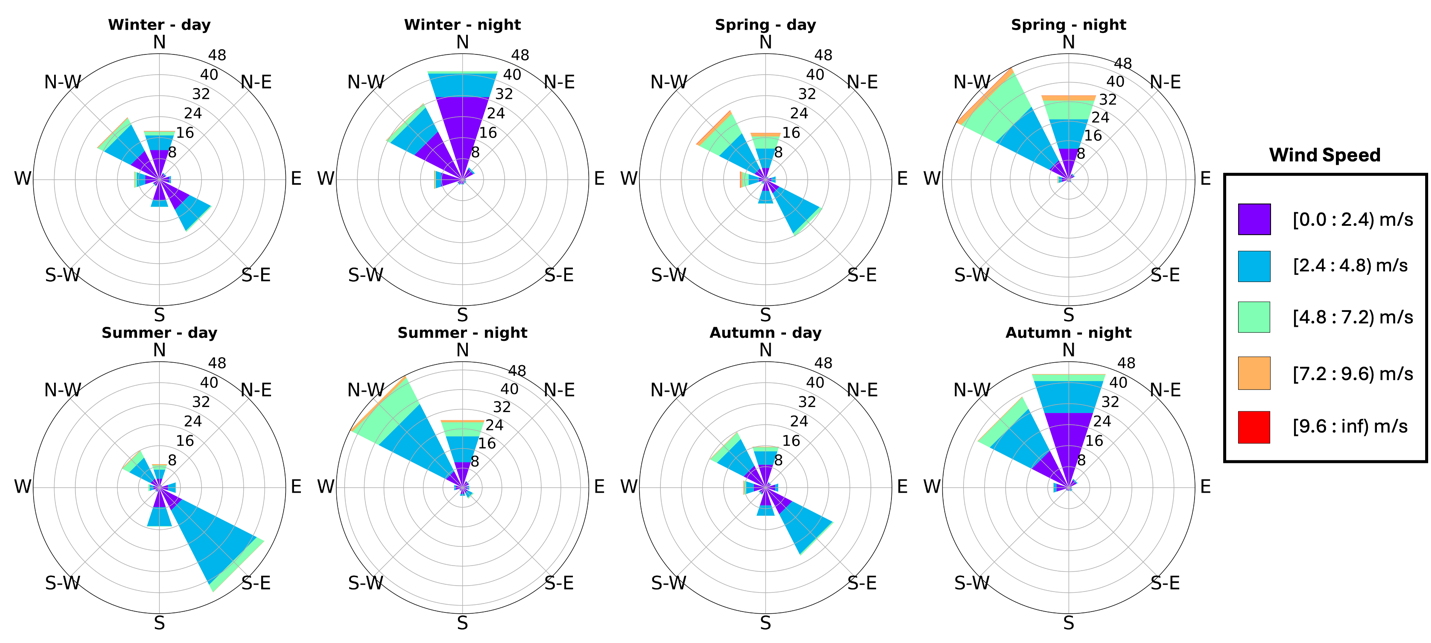


**Figure S2.** Seasonal and diurnal wind patterns are shown for Thermal. Although Thermal is further northwest from Calipatria, influence from the North American Monsoon can be observed in the summer months shown by the southeasterly flow. (Daytime: [06:00 – 18:00]; nighttime: [19:00 – 05:00]).


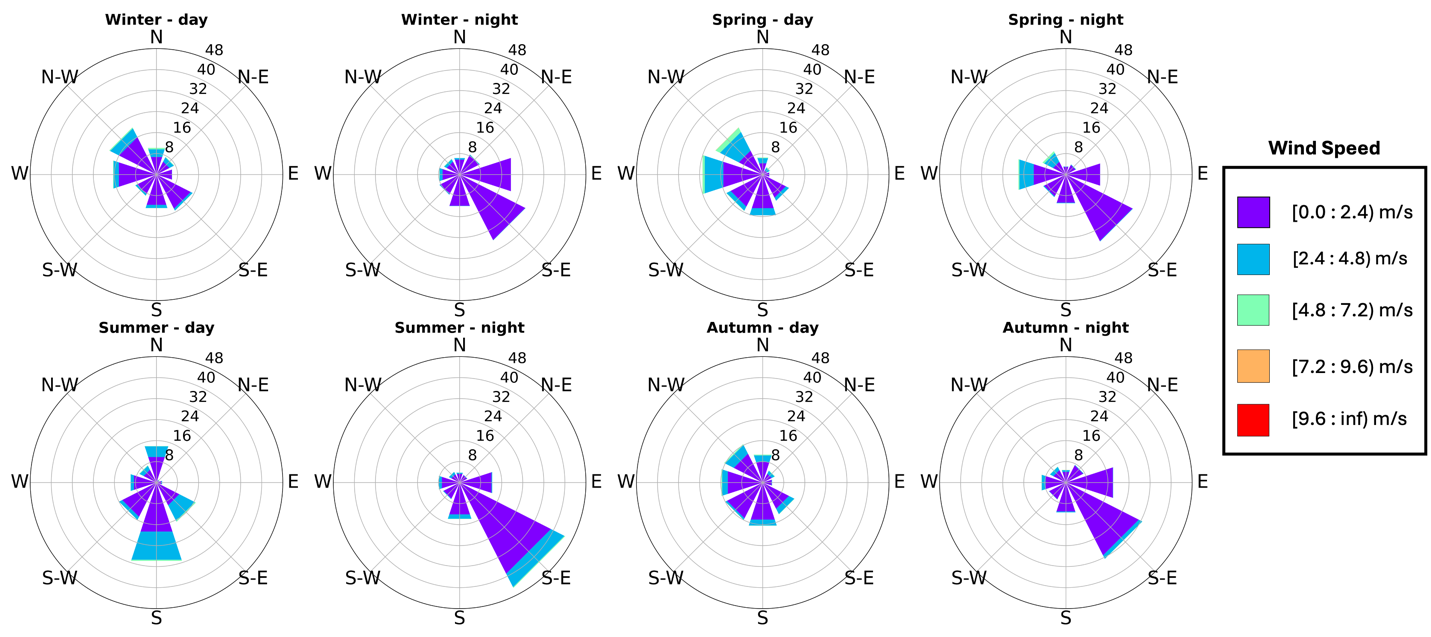


**Figure S3.** Seasonal and diurnal wind patterns are shown for Calipatria. Southerly and southeasterly winds dominate in the summer months due to influence from the North American Monsoon originating from the Gulf of California. (Daytime: [06:00 – 18:00]; nighttime: [19:00 – 05:00]).

**Text S1:** Satellite NO_2_ patterns and regional winds

The general flow throughout the Salton Sea Air Basin (SSAB) is the superposition of three different scales (Figures S2 & S3). At the basin scale there is a consistent lake-breeze circulation coming off the Salton Sea during the day (southeasterlies at Thermal, northwesterlies at Calipatria) and reverse at night. At the continental scale there is a North American monsoon characterized by inflow from the Gulf of California (south-southeasterly) which tends to prevail in the warmest months of July/August and mostly influences the Imperial Valley (south of the Salton Sea). On the synoptic scale the overlying westerlies produce flow through the gaps in the surrounding mountains: particularly pronounced westerlies channeled through the Banning pass upwind of Palm Springs, and westerlies to the south where gaps in the valley's western rim (formed by the Peninsular Mountain Range) allow the synoptic flow to sweep over the Imperial Valley.^1^

Winds in the Coachella Valley are approximately 4 ms^-1^ and the Thermal site lies about 75 km downwind from Banning, which lies at the eastern outflow of urban emissions channeled in from the Los Angeles basin. This represents an advection time scale of about 4.5 hours, similar to a warm season photochemical lifetime of NO_x_.^2^ Winds in the Imperial Valley tend to be weaker and in July/August during the monsoon when flow from Mexicali takes about 5.5 hours to arrive at Calipatria (~50 km, wind speed ~2.5 ms^-1^). Distances from the San Diego/Tijuana urban complex are about 150 km, taking over 10 hours to arrive in the Imperial Valley. This is greater than the photochemical lifetime of NO_x_ even during winter.

The NO_2_ satellite data shows how the NO_x_ at our sampling sites are mostly isolated from large urban sources (Figure 1 & S1). The lake breeze circulation surrounding the Salton Sea likely goes a long way to recirculating emissions within the basin. Moreover, any influence from exogenous urban sources dominated by automobile emissions would tend to bias our measurements to larger δ^15^N characteristic of those sources, making our estimates using only basin emissions a lower limit.

**Table S1**. List of materials needed for denuder preparation and CCSC setup and extraction.

| **Chemical/material** | **Manufacturer** | **Grade/description** |
| --- | --- | --- |
| Potassium hydroxide | Thermo-Fisher Scientific | Flake, 85% |
| Methanol | Thermo-Fisher Scientific | 99.8+%, ACS reagent |
| Guaiacol | Thermo-Fisher Scientific | 99+% |
| Sulfanilaminde | Sigma Aldrich | 99+% |
| N-(1-Naphthyl) ethylenediamine dihydrochloride (NED) | Sigma Aldrich | >98% |
| Vanadium (III) chloride | Sigma Aldrich |  |
| Sodium nitrite | Sigma Aldrich | 99.999% trace metals basis |
| Sodium nitrate | Sigma Aldrich | 99.999% trace metals basis |
| Hydrochloric acid | Thermo-Fisher Scientific | ACS certified |
| Nylon filters | Measurement Technologies Laboratory | 47 mm, 1μm pore spaced, prewashed |
| Whatman filters #1 | Sigma Aldrich |  |
| Mass flow controller | MKS | 10 LPM |

**Text S2**: Detailed CCSC set-up and extraction

Nylon filters (NY47P) were purchased from Measurement Technology Laboratories (MTL). Filters were 46.2 mm in diameter with 1 μm pore size and were pre-washed. Filters were washed again before field-sampling: first, filters were placed in a container filled with MQ-H_2_O and placed on an orbital shaker for 1 hour. Then filters were dried in low heat for 20-30 minutes and stored in a petri-dish. Honeycomb denuders were soaked in 10% HCl for 24 hours, then rinsed with Milli-Q H_2_O and dried with N_2_ gas. Red caps were placed on either end of the denuders for storage until use. For sampling, denuders were coated with 2.5 M potassium hydroxide + 25% by wt. guaiacol. This solution was made fresh each time and 10 mL of this solution was applied to each denuder, first removing a single red cap and evenly distributing the solution among the pores, then re-capping and shaking to distribute the solution evenly. Excess solution was poured into the hazardous waste container and caps were removed so the denuders could dry, then were recapped with clean caps. The rest of the preparation and extraction steps were followed based on the manufacturer’s recommendations.^3^

Immediately after ambient air collections arrived from shipment, denuders were extracted using 30 mL of Milli-Q water and nylon filters were extracted using 20 mL of Milli-Q water. Extracted samples were then filtered via vacuum filtration using a Whatman #1 filter (55 mm). Standards of NaNO_2_ and NaNO_3_ for UV-Vis analysis were prepared from 5 μM to 200 μM to ensure the collected concentrations of analyte were within the range. The coloring agent for the nitrite samples were made differently from the nitrate samples. For nitrite, two solutions were prepared, 10% sulfanilamide in 30% HCl: 70% MQ-H_2_O, and 1% NED in MQ-H_2_O. For UV-Vis analysis, 1 mL of analyte or standard was added to the cuvette, then 25 μL of sulfanilamide solution was added and the cuvette was capped and inverted to mix. The cuvettes sat for 10 minutes, then 25 μL of the NED solution was added and inverted. Solutions were allowed to sit for 10 minutes before analyzing on UV-Vis. For nitrate, the preparation for the coloring solution was prepared as reported in Doane and Horwath, 2003.^4^ After concentrations were determined sufficient for submission to the SIF, nitrite solutions were neutralized using 0.1 M HCl.


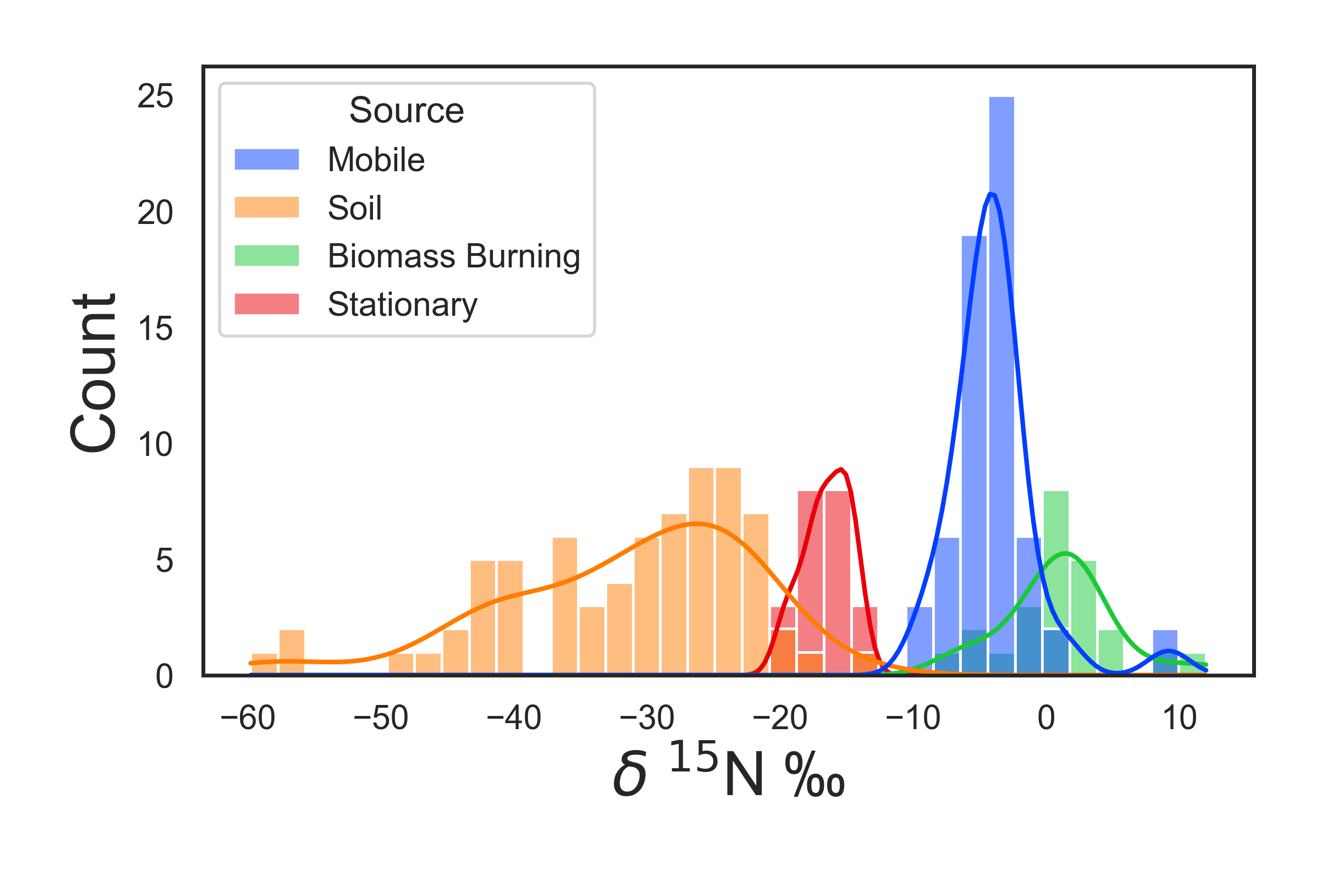


**Figure S4**. Probability distributions for each soil source and their spread of measured isotopic signatures.^5–11^

**Text S3.** Isotopic fractionation calculations for δ^15^N.

N isotopic fractionations are associated with the photostationary equilibrium of O_3_-NO-NO_2_ (Leighton Cycle) and were accounted for using Equations S1-S3 below from Bekker et al., 2023. The influence of δ^15^N fractionation associated with NO_x_ cycling is shown in Equation S1:

$\delta({}^{15}{, {NO}_{x})\approx{}^{15}{\varepsilon\left( \frac{{NO}_{2}}{NO} \right)\times\left( 1-f\left( {NO}_{2} \right) \right)-} \delta({}^{15}N,{NO}_{2})}$ **(S1)**

where δ(^15^N,NO_2_) represents our isotopic measurements from the field study, ^15^ε(NO_2_/NO) is the isotope effect associated with NO conversion to NO_2_, and f(NO_2_) represents the fraction of NO_2_ in NO_x_. Additionally, the ^15^ε(NO_2_/NO) value represents a combination of Leighton Cycle isotope effects (LCIE) and equilibrium isotope effects (EIE), refer to Bekker et al. (2023) for a detailed explanation.^12^ Further, they estimated the relative role of EIE and LCIE in Equation S2 and S3. The isotope effects were modeled in previous studies and these estimates were used in our calculations.

${}^{15}{\varepsilon\left( \frac{{NO}_{2}}{NO} \right)=f_{EIE}({}^{15}{\varepsilon_{EIE})+(1-f_{EIE})({}^{15}{\varepsilon_{LCIE})}}}$ **(S2)**

$f_{EIE}= \frac{k\left( {NO}_{x}-EIE \right)[{NO}_{2}]}{k\left( NO+O_{3} \right)\left[ O_{3} \right]+k\left( {NO}_{x}-EIE \right)[{NO}_{2}]}$ **(S3)**

**Table S2**. Sampling durations at each site during the field study.

| Calipatria | | | Thermal | | |
| --- | --- | --- | --- | --- | --- |
| Start time | Stop time | Duration | Start time | Stop time | Duration |
| 06/16/22 1400 PST | 06/18/22 1900 PST | 53 h |  |  |  |
| 07/20/22 1500 PST | 07/23/22 1000 PST | 67 h | 07/22/22 1400 PST | 07/27/22 1200 PST | 118 h |
| 08/24/22 1400 PST | 08/27/22 0900 PST | 67 h | 08/24/22 1000 PST | 08/30/22 0900 PST | 143 h |
| 09/14/22 1200 PST | 09/19/22 0900 PST | 117 h | 09/19/22 1200 PST | 09/26/22 1000 PST | 166 h |
| 10/14/22 1100 PST | 10/18/22 1100 PST | 96 h | 10/20/22 1000 PST | 10/25/22 0900 PST | 119 h |
| 11/18/22 1300 PST | 11/22/22 1200 PST | 95 h | 11/17/22 1200 PST | 11/21/22 1100 PST | 95 h |
| 12/16/22 1300 PST | 12/21/22 1100 PST | 118 h | 12/15/22 1100 PST | 12/19/22 1200 PST | 97 h |
| 01/20/23 1200 PST | 01/25/23 1100 PST | 119 h | 01/12/23 1100 PST | 01/17/23 0800 PST | 117 h |
| 02/24/23 1100 PST | 02/28/23 1300 PST | 98 h | 02/27/23 0900 PST | 03/01/23 0900 PST | 48 h |
| 04/19/23 1100 PST | 04/23/23 1300 PST | 98 h | 04/12/23 0800 PST | 04/17/23 0900 PST | 121 h |
| Total time sampled | | 928 h  (~39 d) | Total time sampled | | 1024 h  (~43 d) |

**Table S3**. Average meteorology and pollutant concentrations during field sampling in Calipatria (June 2022 – April 2023). Volumetric soil moisture (VSM) was reported from SMAP satellite data). NO_x_, O_3_, and PM_2.5_ data were obtained from the nearest meteorological sites (El Centro, Niland, and Brawley, respectively). See Table S2 for sampling durations. Shaded rows indicate negligible soil NO_2_ observed.

| Month | T_max_ (°C) | O_3_ (ppb) | NO_x_ (ppb) | PM_2.5_ (μg/m^3^) | PM_10_  (μg/m^3^) | Scalar ws (m/s) | wd | RH (%) | Specific humidity | Precipitation  (cm) | VSM  (m^3^/m^3^) |
| --- | --- | --- | --- | --- | --- | --- | --- | --- | --- | --- | --- |
| Jun | 41.2 | 40.4 | 3.6 | 34.6 | 82.9 | 3.9 | 220° | 16.5 | 0.004 | 0 | 0.084 |
| Jul | 44.2 | 28.0 | 5.7 | 6.5 | 56.3 | 2.7 | 132° | 30.5 | 0.011 | 0 | 0.088 |
| Aug | 43.8 | 28.5 | 5.6 | 5.6 | 35.9 | 2.7 | 135° | 36.5 | 0.013 | 0.02 | 0.103 |
| Sep | 38.2 | 28.1 | 8.4 | 4.3 | 40.1 | 2.0 | 155° | 37.0 | 0.008 | 0 | 0.116 |
| Oct | 32.9 | 21.7 | 6.7 | 3.7 | 21.2 | 1.8 | 140° | 59.2 | 0.011 | 3.39 | 0.154 |
| Nov | 23.3 | 28.7 | 8.3 | 3.9 | 23.6 | 2.3 | 0° | 32.3 | 0.003 | 0 | 0.116 |
| Dec | 18.9 | 23.1 | 13.0 | 6.4 | 21.8 | 1.8 | 109° | 42.5 | 0.003 | 1.47 | 0.117 |
| Jan | 19.2 | 20.1 | 9.8 | 5.5 | 19.0 | 2.7 | 337° | 31.8 | 0.002 | 0.25 | 0.151 |
| Feb | 18.8 | 39.0 | 3.6 | 1.0 | 9.8 | 4.5 | 233° | 51.8 | 0.005 | 0.11 | 0.152 |
| Apr | 35.1 | 38.1 | 4.2 | 8.2 | 32.4 | 2.5 | 205° | 27.7 | 0.004 | 0.05 | 0.108 |
| Avg | 31.5 | 29.7 | 6.9 | 8.0 | 34.2 | 2.7 | 163° | 36.7 | 0.007 | (sum) 5.29 | 0.119 |

**Table S4**. Average meteorology and pollutant concentrations during field sampling in Thermal (July 2022 – April 2023). O_3_, NO_x_, and PM_2.5_ were obtained from the nearest monitoring locations (Palm Springs and Brawley). ND denotes no data. Volumetric soil moisture (VSM) was reported from SMAP satellite data). See Table S2 for sampling durations. Shaded rows indicate negligible soil NO_x_ observed.

| Month | T_max_ (°C) | O_3_ (ppb) | NO_x_ (ppb) | PM_2.5_ (μg/m^3^) | PM_10_  (μg/m^3^) | Scalar ws (m/s) | wd | RH (%) | Specific humidity | Precipitation  (cm) | VSM  (m^3^/m^3^) |
| --- | --- | --- | --- | --- | --- | --- | --- | --- | --- | --- | --- |
| Jul | 40.2 | 54.7 | 5.5 | 9.5 | ND | 2.7 | 139° | 32.6 | 0.011 | 0 | 0.035 |
| Aug | 40.5 | 50.4 | 4.3 | 2.7 | 31.9 | 3.1 | 347° | 27.4 | 0.009 | 0.01 | 0.043 |
| Sep | 38.9 | 43.5 | 4.9 | 5.2 | 20.1 | 2.7 | 326° | 24.3 | 0.007 | 0.01 | 0.051 |
| Oct | 29.0 | 41.2 | 5.6 | 6.9 | ND | 2.9 | 303° | 28.4 | 0.005 | 0.24 | 0.063 |
| Nov | 23.4 | 34.7 | 10.6 | 3.4 | 15.8 | 2.3 | 310° | 29.6 | 0.002 | 0.62 | 0.061 |
| Dec | 18.1 | 24.3 | 14.6 | 5.6 | 15.0 | 1.3 | 307° | 47.3 | 0.003 | 0.30 | 0.063 |
| Jan | 19.2 | 28.2 | 10.2 | 6.4 | 11.6 | 2.5 | 328° | 60.4 | 0.005 | 0.05 | 0.105 |
| Feb | 17.4 | 36.9 | 6.5 | ND | 4.7 | 3.1 | 297° | 51.6 | 0.005 | 0 | 0.122 |
| Apr | 29.9 | 51.3 | 3.9 | 14.7 | 69.5 | 4.9 | 328° | 37.7 | 0.006 | 0 | 0.082 |
| Avg | 28.5 | 38.4 | 7.3 | 6.04 | 18.7 | 2.8 | 317° | 37.7 | 0.006 | (sum) 1.23 | 0.069 |

**Table S5**. Climatology data for Calipatria, or nearest Imperial County met site* (Niland, Brawley, El Centro).

| Month | T_max­_ (°C) | O_3_ (ppb)* | NO_x_ (ppb)* | PM_2.5_  (μg/m^3^)* | PM_10_  (μg/m^3^) | Scalar ws (m/s) | wd | RH (%) | Specific Humidity | Precipitation  (cm) |
| --- | --- | --- | --- | --- | --- | --- | --- | --- | --- | --- |
| Jan | 21.3 | 27.7 | 17.6 | 4.0 | 25.1 | 1.5 | 292° | 51.6 | 0.004 | 0.69 |
| Feb | 23.0 | 35.0 | 13.5 | 4.4 | 30.5 | 1.6 | 269° | 43.5 | 0.004 | 0.68 |
| Mar | 26.4 | 41.2 | 9.1 | 4.6 | 36.4 | 1.8 | 277° | 40.5 | 0.005 | 0.58 |
| Apr | 29.6 | 46.3 | 6.1 | 7.1 | 50.3 | 2.0 | 269° | 33.8 | 0.005 | 0.19 |
| May | 33.3 | 49.1 | 4.3 | 8.3 | 55.2 | 2.1 | 247° | 31.5 | 0.006 | 0.18 |
| Jun | 38.9 | 47.4 | 5.1 | 8.9 | 56.6 | 2.0 | 191° | 27.5 | 0.007 | 0.02 |
| Jul | 41.0 | 39.2 | 4.8 | 8.6 | 55.7 | 2.3 | 159° | 31.7 | 0.010 | 0.14 |
| Aug | 41.0 | 39.6 | 5.1 | 7.5 | 52.3 | 2.1 | 166° | 34.4 | 0.011 | 1.27 |
| Sep | 38.3 | 36.8 | 6.7 | 7.5 | 49.6 | 1.8 | 178° | 36.3 | 0.010 | 0.61 |
| Oct | 32.1 | 34.6 | 9.4 | 6.8 | 46.5 | 1.7 | 268° | 33.9 | 0.006 | 0.56 |
| Nov | 25.7 | 29.8 | 14.9 | 5.0 | 39.5 | 1.5 | 283° | 42.5 | 0.005 | 0.33 |
| Dec | 20.0 | 25.6 | 17.7 | 4.0 | 26.8 | 1.5 | 315° | 48.4 | 0.004 | 0.86 |
| Avg | 29.3 | 38.5 | 9.5 | 6.6 | 44.5 | 1.8 | 244 | 38.4 | 0.007 | (sum) 6.11 |

**Table S6**. Climatology data for Thermal, or nearest air quality site (Palm Springs*). RH data was not available for Thermal, so the nearby Torres-Martinez^⊕^ site was substituted.

| Month | T_max­_ (°C) | O_3_ (ppb)* | NO_x_ (ppb)* | PM_2.5_  (μg/m^3^) | PM_10_  (μg/m^3^) | Scalar ws (m/s) | wd | RH (%)^⊕^ | Specific Humidity | Precipitation  (cm) |
| --- | --- | --- | --- | --- | --- | --- | --- | --- | --- | --- |
| Jan | 22.0 | 37.8 | 15.7 | 4.3 | 15.5 | 1.9 | 327° | 56.3 | 0.004 | 1.89 |
| Feb | 23.7 | 46.2 | 11.5 | 4.4 | 21.4 | 2.4 | 327° | 50.5 | 0.004 | 0.91 |
| Mar | 27.6 | 53.4 | 8.0 | 4.4 | 25.4 | 3.3 | 325° | 40.0 | 0.005 | 0.69 |
| Apr | 31.3 | 61.5 | 5.6 | 6.7 | 42.3 | 4.1 | 327° | 35.4 | 0.005 | 0.22 |
| May | 34.7 | 65.8 | 4.3 | 7.2 | 42.1 | 4.4 | 329° | 34.9 | 0.006 | 0.02 |
| Jun | 40.0 | 71.9 | 4.6 | 8.6 | 46.6 | 3.8 | 331° | 33.7 | 0.007 | 0 |
| Jul | 41.9 | 68.6 | 4.6 | 8.1 | 43.6 | 3.3 | 342° | 37.9 | 0.010 | 0.34 |
| Aug | 41.8 | 67.8 | 5.2 | 9.0 | 36.3 | 3.1 | 342° | 37.2 | 0.010 | 0.18 |
| Sep | 38.7 | 58.6 | 6.1 | 8.4 | 36.1 | 2.8 | 339° | 41.2 | 0.008 | 0.76 |
| Oct | 32.6 | 52.0 | 8.4 | 6.7 | 31.8 | 2.7 | 331° | 41.0 | 0.006 | 0.54 |
| Nov | 26.3 | 42.4 | 12.8 | 5.5 | 29.9 | 2.1 | 327° | 50.5 | 0.005 | 0.19 |
| Dec | 20.7 | 35.8 | 15.6 | 5.3 | 24.8 | 1.9 | 326° | 55.1 | 0.004 | 1.08 |
| Avg | 30.1 | 54.5 | 8.5 | 6.9 | 31.3 | 3.2 | 332 | 40.3 | 0.006 | (sum) 6.82 |


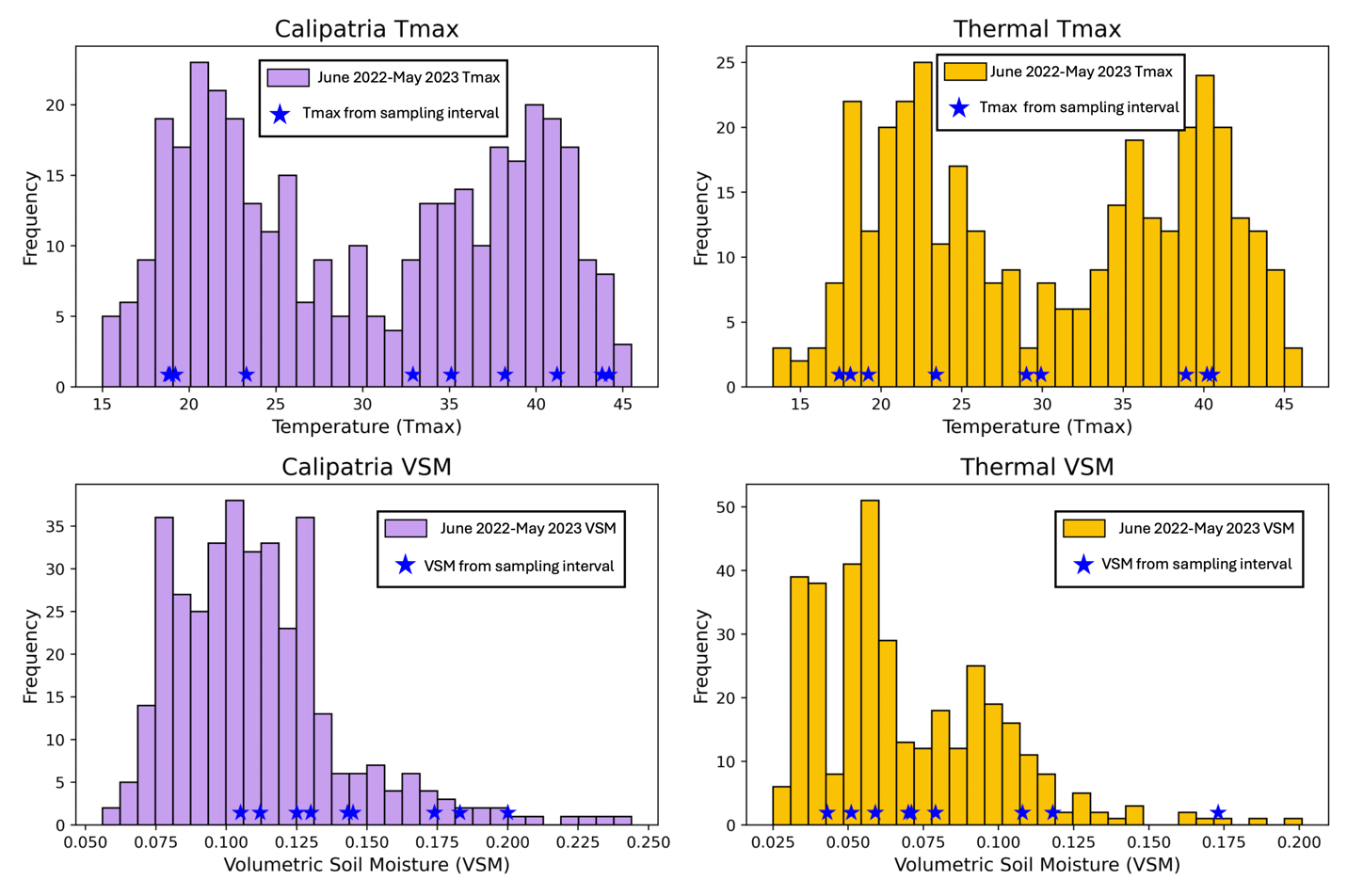


**Figure S5**. Histograms of observed T_max_ and VSM June 2022-May 2023 for Calipatria/Imperial Valley (purple) and Thermal/ Coachella Valley (gold) are reported. Values from our sampling intervals are marked with blue stars.

**Table S7.** Average meteorological and pollutant parameters and standard deviation reported for our sampling periods and for the sampling year (June 2022-May 2023). Note that the reported VSM values are for 16:30 to correspond with the time in which temperature is at its maximum.

|  | **Average** | T_max_ | O_3_ (ppb) | NO_x_ (ppb) | PM_2.5_ (μg/m^3^) | PM_10_  (μg/m^3^) | Scalar ws (m/s) | RH (%) | VSM  (m^3^/m^3^) |
| --- | --- | --- | --- | --- | --- | --- | --- | --- | --- |
| Calipatria/  Imperial Valley | **Sampling Interval** | 31.6 ± 10 | 38.1 ± 8 | 4.2 ± 3 | 8.2 ± 12 | 32.4 ± 22 | 2.5 ± 0.9 | 36.7 ± 12 | 0.12 ± 0.03 |
|  | **Annual** | 30.0 ± 9 | 30.7 ± 12 | 7.8 ± 5 | 7.8 ± 7 | 41.8 ± 41 | 2.1 ± 1 | 49.1 ± 12 | 0.11 ± 0.03 |
| Thermal/ Coachella Valley | **Sampling Interval** | 28.5 ± 10 | 38.4 ± 11 | 7.3 ± 4 | 6.1 ± 4 | 18.7 ± 22 | 2.8 ± 1 | 37.7 ± 13 | 0.07 ± 0.03 |
|  | **Annual** | 30.4 ± 9 | 43.0 ± 14 | 6.9 ± 4 | 7.8 ± 7 | 29.3 ± 39 | 3.0 ± 2 | 40.5 ± 14 | 0.07 ± 0.03 |

**References**

1. Evan, A. T. Downslope Winds and Dust Storms in the Salton Basin. *Mon. Weather Rev.* **147**, 2387–2402 (2019).

2. Beirle, S., Boersma, K. F., Platt, U., Lawrence, M. G. & Wagner, T. Megacity Emissions and Lifetimes of Nitrogen Oxides Probed from Space. *Science* **333**, 1737–1739 (2011).

3. Thermo Scientific. Partisol Model 2300: Speciation Sampler Operation Manual. (2005).

4. Doane, T. A. & Horwáth, W. R. Spectrophotometric Determination of Nitrate with a Single Reagent. *Anal. Lett.* **36**, 2713–2722 (2003).

5. Fibiger, D. L. & Hastings, M. G. First Measurements of the Nitrogen Isotopic Composition of NO *_x_* from Biomass Burning. *Environ. Sci. Technol.* **50**, 11569–11574 (2016).

6. Li, D. & Wang, X. Nitrogen isotopic signature of soil-released nitric oxide (NO) after fertilizer application. *Atmos. Environ.* **42**, 4747–4754 (2008).

7. Miller, D. J., Wojtal, P. K., Clark, S. C. & Hastings, M. G. Vehicle NOx emission plume isotopic signatures: Spatial variability across the eastern United States. *J. Geophys. Res. Atmospheres* **122**, 4698–4717 (2017).

8. Miller, D. J. *et al.* Isotopic Composition of In Situ Soil NO _x_ Emissions in Manure‐Fertilized Cropland. *Geophys. Res. Lett.* **45**, (2018).

9. Walters, W. W., Goodwin, S. R. & Michalski, G. Nitrogen Stable Isotope Composition (δ15N) of Vehicle-Emitted NOx. *Environ. Sci. Technol.* **49**, 2278–2285 (2015).

10. Walters, W. W., Tharp, B. D., Fang, H. & Michalski, G. Nitrogen Isotope Composition of Thermally Produced NOx from Various Fossil-Fuel Combustion Sources. *Environ. Sci. Technol.* **49**, 11363–11371 (2015).

11. Yu, Z. & Elliott, E. M. Novel Method for Nitrogen Isotopic Analysis of Soil-Emitted Nitric Oxide. *Environ. Sci. Technol.* **51**, 6268–6278 (2017).

12. Bekker, C., Walters, W. W., Murray, L. T. & Hastings, M. G. Nitrate chemistry in the northeast US – Part 1: Nitrogen isotope seasonality tracks nitrate formation chemistry. *Atmospheric Chem. Phys.* **23**, 4185–4201 (2023).
